# Supplementary material for: Virulence Profiles of Wild-Type, P.1 and Delta SARS-CoV-2 Variants in K18-hACE2 Transgenic Mice
Source: Viruses. 2023 Apr 19;15(4):999. doi: 10.3390/v15040999 (PMC10146242; doi:10.3390/v15040999)
Supplement: Supplementary file 1 [file viruses-15-00999-s001.zip › Table S1.pdf]

Table S1: The gender analysis of weight and clinical score per day post-infection for K18-hACE2 transgenic mice infected intranasally with 10^5 PFU of the Wt strain or the P.1 and Delta variants.

	**Weight**			**Clinical score**		
	**1 dpi**					
	Wt	P.1	Delta	Wt	P.1	Delta
**Male X Female**	>0,9999	>0,9999	>0,9999	All the values are the same, so no statistical test is needed.		
	**2 dpi**					
	>0,9999	>0,9999	>0,9999	>0,9999	>0,9999	>0,9999
	**3 dpi**					
	>0,9999	>0,9999	>0,9999	>0,9999	>0,9999	>0,9999
	**4 dpi**					
	>0,9999	>0,9999	>0,9999	>0,9999	>0,9999	>0,9999
	**5 dpi**					
	>0,9999	>0,9999	>0,9999	>0,9999	>0,9999	>0,9999
	**6 dpi**					
	>0,9999	X	>0,9999	>0,9999	X	>0,9999
	**7 dpi**					
	>0,9999	X	>0,9999	0,8728	X	0,8256

Test performed by One-way ANOVA, with Bonferroni multivariance analysis for parametric variables, and Kruskal Wallis with Dunn´s post hoc test for non parametric variables, using GraphPad Prism 8.0 version.
