# Supplementary material for: Virulence Profiles of Wild-Type, P.1 and Delta SARS-CoV-2 Variants in K18-hACE2 Transgenic Mice
Source: Viruses. 2023 Apr 19;15(4):999. doi: 10.3390/v15040999 (PMC10146242; doi:10.3390/v15040999)
Supplement: Supplementary file 1 [file viruses-15-00999-s001.zip › Figure S2.pdf]

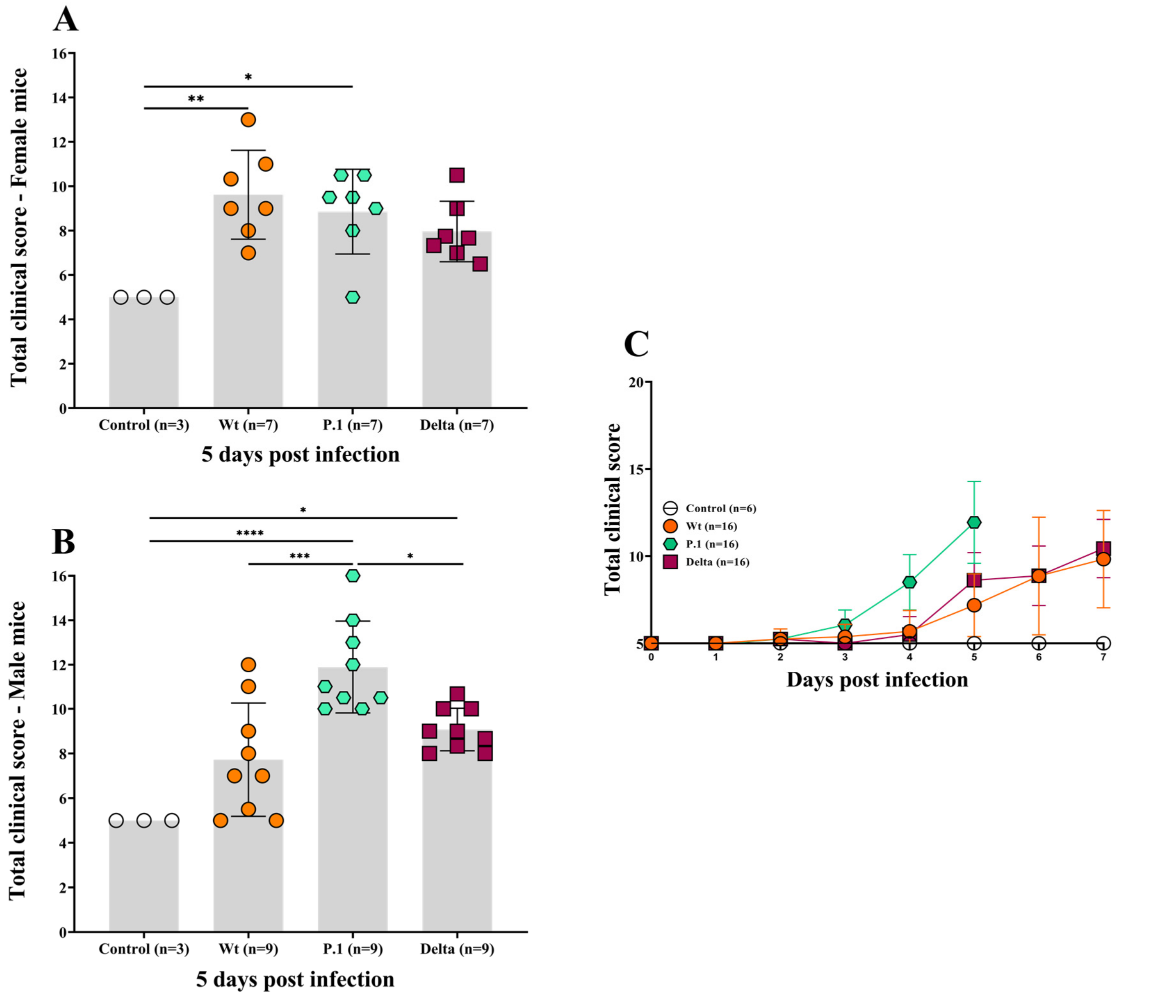

Figure S2. The evaluation of clinical manifestations in female (**A**) and male (**B**) mice. Clinical score kinetics per day post-infection of K18-hACE2 transgenic mice infected intranasally with 10^5 PFU of the Wt strain and the P.1 and Delta variants (**C**).
