# Supplementary material for: Virulence Profiles of Wild-Type, P.1 and Delta SARS-CoV-2 Variants in K18-hACE2 Transgenic Mice
Source: Viruses. 2023 Apr 19;15(4):999. doi: 10.3390/v15040999 (PMC10146242; doi:10.3390/v15040999)
Supplement: Supplementary file 1 [file viruses-15-00999-s001.zip › Figure S3.pdf]

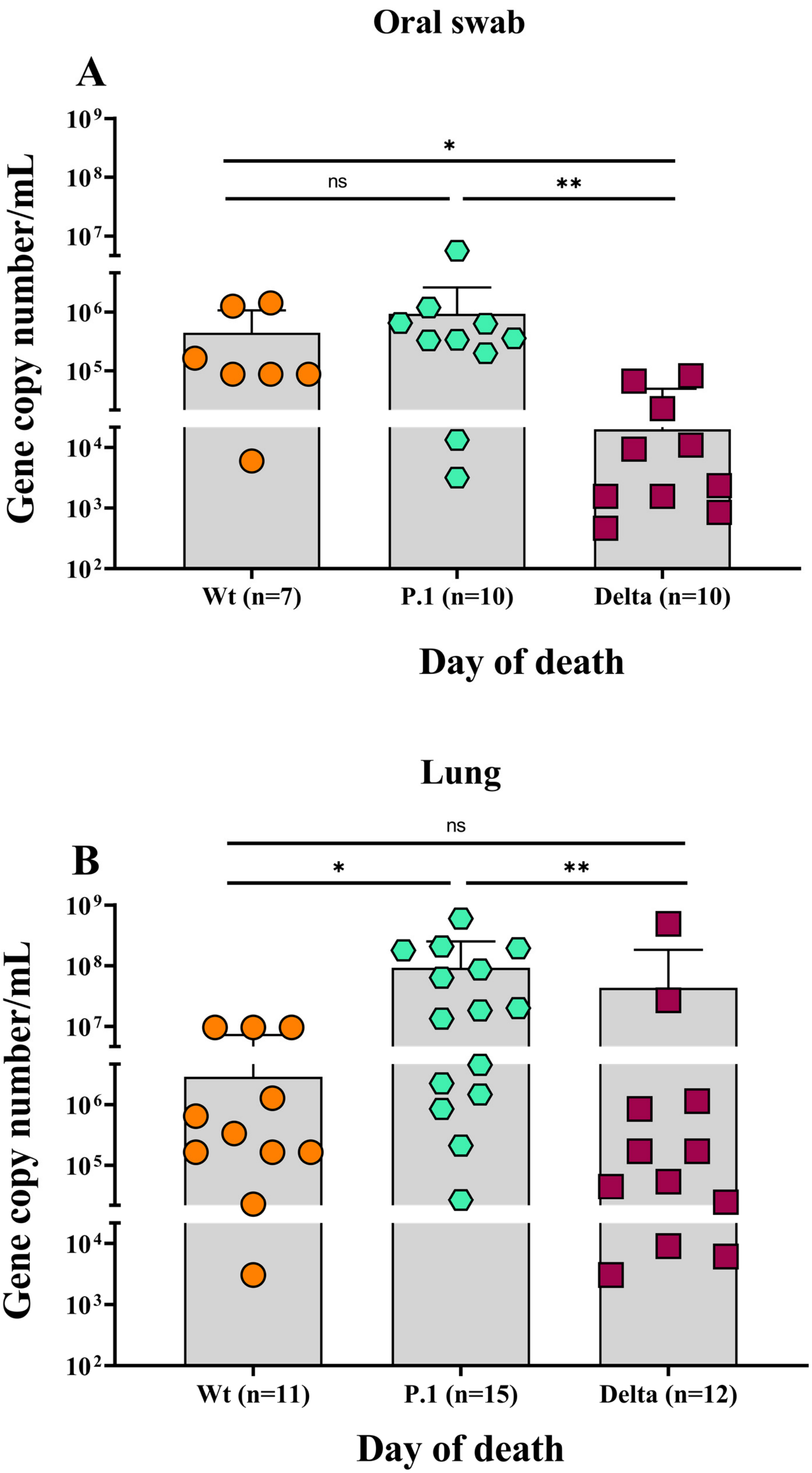

Figure S3. The quantitative detection and viral load analysis in the lung and oral swabs of K18-hACE2 mice infected with the Wt strain or the P.1 and Delta SARS-CoV-2 variants. Viral load detection by RT-qPCR in lung (**A**) and oral (**B**) swabs at the day of death (timepoint or endpoint) from K18-hACE2 mice infected with the Wt strain or the P.1 and Delta variants. Test performed by Kruskal–Wallis test followed by Dunn's post hoc test with the use of GraphPad Prism 8.0, where $p < 0.05$.
